# Descriptive study on risk of increased morbidity of schistosomiasis and graft loss after liver transplantation

**DOI:** 10.1590/0037-8682-0097-2024

**Published:** 2024-07-29

**Authors:** Carlos Graeff-Teixeira, Clairton Marcolongo-Pereira, Betina Bolina Kersanach, Stefan Michael Geiger, Deborah Negrão-Correa

**Affiliations:** 1 Universidade Federal do Espírito Santo, Centro de Ciências da Saúde, Departamento de Patologia e Núcleo de Doenças Infecciosas, Vitória, ES, Brasil.; 2 Centro Universitário do Espírito Santo, Faculdade de Medicina, Colatina, ES, Brasil.; 3 Instituto de Ciências Biológicas, Universidade Federal de Minas Gerais, Departamento de Parasitologia, Belo Horizonte, MG, Brasil.; 4 Instituto de Ciências Biológicas, Universidade Federal de Minas Gerais, Laboratório de Esquistossomose e Imuno-helmintologia - Departamento de Parasitologia, Belo Horizonte, MG, Brasil.

**Keywords:** Liver, Transplantation, Schistosoma mansoni, *Schistosoma* spp, Schistosomiasis

## Abstract

Solid-organ transplantation procedures have witnessed a surge in frequency. Consequently, increased attention to associated infections and their impact on graft success is warranted. The liver is the principal target for infection by the flatworm *Schistosoma mansoni*. Hence, rigorous screening protocols for this parasite should be implemented for liver transplantation donors and recipients. This study investigated the risks posed by schistosomiasis-infected liver tissues for successful liver transplantation (LT), considering donors and recipients, by analyzing reported cases. Among the 43 patients undergoing LT (donors = 19; recipients = 24), 32 were infected with *S. mansoni*, five were infected with other *Schistosoma* species, and no identification was made in four patients. Reported follow-up periods ranged from 1 to 132 months, and all patients achieved successful recovery. As these helminths do not replicate in their vertebrate hosts, immunosuppressive treatment is not expected to promote increased morbidity or reactivation. Moreover, suspected or confirmed schistosomiasis infections often have a benign course, and generally, should not prevent LT. The available literature was reviewed and a provisional screening protocol has been proposed.

## INTRODUCTION

Owing to the rising demand for organ transplantation, imbalances in the available supply of donations have emerged^1^. In this context, expanded donor criteria (ECD), or consideration of “marginal” or “less optimal donors”, have been proposed^1^ ^-^ ^3^. The use of ECD is particularly relevant to candidates for transplantation affected by more severe clinical conditions and has led to the consideration of schistosomiasis-infected organs for transplantation^4^ ^,^ ^5^. 

Infectious complications are most frequently caused by bacteria and contribute to poor outcomes in 75% of transplants and 30% of graft losses^6^ ^-^ ^8^. The least frequent group of infectious agents complicating transplantations is parasites, and infections by helminths are even rarer^9^ ^,^ ^10^. For example, helminthic infections are estimated to affect less than 4% of patients with transplants^4^ ^,^ ^8^ ^,^ ^11^ ^-^ ^13^.

The kidney is the most frequently transplanted organ, followed by the liver and other solid organs. Schistosomiasis associated with kidney transplantation is not considered an important factor for poor prognosis as observed in several studies involving a considerable number of patients^14^ ^-^ ^16^. However, there are concerns regarding lower urinary tract damage related to infections by *S. haematobium*, *S. mansoni*, and other schistosomes. Heart and bone marrow transplantations are also very rarely associated with schistosomiasis, and there is no evidence of increased morbidity due to parasitic infection^17^ ^,^ ^18^. Here, we highlight *Schistosoma* species that reside within the portal-mesenteric venous vessels, thereby representing a direct potential cause of failed liver transplantation (LT).

Schistosomiasis is caused by parasitic trematodes of the genus *Schistosoma* ^19^. Currently, schistosomiasis affects over 220 million people living in endemic areas of Africa, Asia, and Latin America^20^ ^-^ ^24^. Three species of the genus *Schistosoma* are important causes of human diseases. *S. mansoni* and *S. japonicum* mainly affect the gastrointestinal tract, while *S. haematobium* affects the urinary tract. 

The larvae that can infect mammalian hosts are released from snails into water collections, where they come into contact with the definitive host and penetrate through the skin. Upon gaining access to blood circulation, larvae migrate to reach maturity in either the upper mesenteric-portal venous system (*S. mansoni* and *S. japonicum*) or in the lower mesenteric and bladder venous vessels (*S. haemtobium*) of a vertebrate host. Sexual reproduction of adult worms and the resulting oviposition occur inside the venous vessels. A portion of the eggs is also released with feces (*S. mansoni* and *S. japonicum*) or urine (*S. haematobium*) to complete the life cycle of the parasite. 

We reviewed the risks of increased morbidity and graft loss associated with schistosomiasis after LT. We also propose provisional diagnostic and treatment recommendations for this rare association.

## METHODS

A review of PubMed, Google Scholar, Latin American and Caribbean Health Sciences Literature, and Cochrane databases was performed on December 18, 2023, using the keywords: “transplantation” AND “schistosomiasis”. The searches covered publications from the 1970’s to 2023. Titles and abstracts of 218 publications identified in the review were examined. A total of 32 publications were subsequently subjected to a full review of the manuscript. Finally, 19 case reports of LT associated with schistosomiasis were selected for analysis when at least two of the following inclusion criteria were present: 1) identification of infected individual: donor or recipient, 2) diagnostic methods were used to detect schistosomiasis, and 3) recipient follow-up and clinical outcomes. At any stage of the review, reports not addressing schistosomiasis-associated LTs were excluded.

## RESULTS

A total of 43 cases reported on between 2003 and 2022 described schistosomiasis-infected LT recipients (n = 24) and donors (n = 19) (Table 1).

All infected recipients had an uneventful early post-transplantation period. In addition, there was no convincing evidence that clinical manifestations or laboratory test abnormalities could be attributed to schistosomiasis. Any clinical or laboratory test abnormality was considered as an indicator of morbidity, requiring careful investigation in a few cases. Abnormal liver enzyme levels were only transiently detected in three patients, demonstrating an active granulomatous reaction and *S.mansoni* eggs^25^ ^,^ ^26^. In two recipients and one infected graft, morbidity caused by parasitic infection was suspected but not confirmed^31^ ^,^ ^33^ ^,^ ^38^. For the two infected recipients, late-stage epithelioid and fibrotic granulomas, and eggs were detected in follow-up biopsies performed 8 months and 11 years after surgery, respectively; however, organ dysfunction was not detected^31^ ^,^ ^38^. Regarding infected grafts, Vincenzi et al.^33^ did not rule out the possibility that schistosomiasis contributed to graft loss in their series of six LTs.

*S. mansoni* was detected in 34 of the 43 cases reviewed (Table 1). In addition, *S. japonicum* was suspected by Patel et al.^37^ in an individual from the Philippines, and confirmed by DNA sequencing in another patient^39^. *S. haematobium* alone^38^ or a mixed infection with *S. mansoni* ^32^ was suspected in two patients. However, these etiological diagnoses were based on histological sections, and the exact identification of the parasite species, according to the morphology and position of the egg spine, cannot always be determined in these types of sections. Moreover, localization of eggs in intestinal and hepatic tissues is not typically observed for *S. haematobium*. Consequently, it is hypothesized that *S. mansoni* was the probable etiological agent based on the report by Kotton et al.^32^. In four reports, the species involved could not be identified (Table 1). 

Follow-up periods were described for 30 of the 43 patients reported and varied from 1 month to 132 months. Moreover, approximately half of the follow-up periods were less than 14 months with a median follow-up period of 12 months (Table 1).

**TABLE 1:** Diagnosis, treatment and follow up of 43 patients with schistosomiasis associated with liver transplantation, from 2003 to 2022.

Reference (year)	Number of Cases	Source of infection	Diagnosis of schistosomiasis	PZQ* treatment before/after liver transplantation	PZQ dosages	Evidence for increased morbidity with schistosomiasis in recipients	Time of follow up (months)
5 **(2003)**	1	Donor	ND**	Before, 5 and 24 months	ND	No	7
27 **(2005)**	1	Donor	Liver biopsy *S.mansoni*	After 2 days repeated 15 days	ND	No	6
28 **(2005)**	1	Recipient	ND	ND	ND	No	ND
29 **(2005)**	2	Recipient 1	Gastric biopsy & serology (ND)	After 6 weeks	40 mg/Kg (1 dosis)	No	18
		Recipient 2	Liver biopsy *S.mansoni*	Before ND time and After 1 year		No	12
30 **(2006)**	3	Donor 1	Liver biopsy & serology	After 1 month	40 mg/Kg (div 2)	No	4 ***
		Donor 2	(MAMA-ELISA) *S.mansoni*	After 1 month		No	7 ***
		Donor 3		After 1 month		No	22 ***
24 **(2006)**	1	Recipient	Liver biopsy & serology, probably *S.mansoni*	After 6 months	60 mg/Kg (1 dosis)	Yes, raised liver enzymes	ND
31 **(2007)**	1	Recipient	Pré-LT: ND	Before LT, ND	ND	Suspected #	132 ****
			Post-LT: intestinal biopsy	time			
			Probably *S.mekongi*				
32 **(2009)**	1	Donor	Intestinal biopsy, brain-dead donor	After 1, 2, 3, 18, 42 days	40 mg/Kg	No	Not informed
					(div 2)		
					(recipient)		
			Suspected *S. haematobium*				
33 **(2011)**	6	Donor 1	Liver biopsy	Living donors were treated	Total 80 mg/Kg	No	56
			*S.mansoni*				
		Donor 2	Liver biopsy	Untreated recipients	20mg/Kg bid for 2 days	No	14
			*S.mansoni*				
		Donor 3	Liver biopsy			No	35
			*S.mansoni*				
		Donor 4	Liver biopsy			No	31
			*S.mansoni*				
		Donor 5	Liver biopsy			No	29
			*S.mansoni*				
		Donor 6	Liver biopsy			Suspected ##	ND
			*S.mansoni*				
34 **(2012)**	1	Recipient	Liver biopsy	ND	ND	ND	ND
10 **(2012)**	2	Donor 1	Liver biopsy	Untreated	Untreated	No, died (sepsis)	0.5 *****
			*S.mansoni*			14 days AFT	
		Donor 2	Eggs in stools	Before LT, time was not informed	ND	No	24 ****
			*S.mansoni*				
35 **(2013)**	2	Recipient 1	Liver biopsy	ND	ND	No	ND
			*S.mansoni*				
		Recipient 2	Liver biopsy	ND	ND	No	ND
			*S.mansoni*				
36 **(2013)**	1	Donor	Liver biopsy	After 19 days	ND	No	ND
			*S.mansoni*				
37 **(2015)**	1	Donor	Liver biopsy	Untreated	untreated	No	ND
			*S.japonicum*				
12 **(2015)**	14	Recipient	Liver histology & serology (COPT)	Before LT	15mg/Kg	No	12-180 ****
			*S.mansoni*	Repeated with positive serology	40 mg/Kg div 2		
38 **(2018)**	1	Recipient	Liver biopsy & serology (AWE-WB), antigen detection (POC-CCA) suspected *S.haematobium*	After 8 months	Repeated after 21 days	Suspected ###	ND
39 **(2019)**	1	Recipient	Liver and intestines histology	Before, 6 yearly treatment and After 1 year	ND	No	24 ****
			Serology: MAMA				
			ELISA				
			DNA sequencing: *S.japonicum*				
40 **(2019)**	1	Donor	Liver biopsy	Untreated	ND	No	ND
			*S.mansoni*				
26 **(2022)**	2	Donor	Liver biopsy	After 18 months	50 mg/Kg	Yes, raised liver enzymes and active granulomas and live eggs	ND
			*S.mansoni*		(1 dosis)		
		Donor	Liver biopsy	After 2 months	50 mg/Kg (1 dosis)		ND
			*S.mansoni*				

*PZQ, praziquantel; **ND, Not Described; Time of follow-up: ***originally expressed, respectively as 20, 35 and 100 weeks, transformed to months by dividing each time length in weeks by 4 weeks; ****originally expressed in years (multiplied by 12 months); *****originally described in days (divided by 30 days); #abnormal transaminases and liver biopsy with calcified eggs and late stage fibrotic granulomas; ##graft loss after 3 days, no mention of lesions attributable to schistosomiasis; ###abnormal transaminases and epithelioid granuloma.

## DISCUSSION

### ● Identification of active schistosomiasis infection

Both transplant donors and recipients underwent a diagnostic test before surgery to evaluate the presence of active schistosomiasis. Eggs were detected in the stool, urine, or tissues. However, diagnostic tests based on parasitological methods lack adequate sensitivity for identifying mild infections, a condition that is becoming increasingly frequent in most endemic areas^41^ ^-^ ^43^. In addition, nucleic acid and antigen detection methods are either not widely available or have not been extensively evaluated but would certainly be valuable diagnostic alternatives that could be applied, at least on an individual basis^44^ ^-^ ^46^. Therefore, schistosomiasis associated with LT during the present review was mainly diagnosed based on histological sections, although one patient was diagnosed with eggs in the stool^10^. Indirect diagnostic methods include serology, the circumoval precipitation test^11^, IgG-ELISA with microsomal antigens (MAMA)^30^ ^,^ ^39^, and Western Blot with adult worm extract^38^. These methods detect the presence of parasite-specific antibodies in infected individuals; however, they can also produce false-positive results because of shared and cross-reacting helminth antigens or immunological memory induced during past infections^47^ ^-^ ^49^ (Table 1). To date, the combination of inadequate testing performance and consequent misinterpretation of serological tests has prevented the release of a definitive recommendation regarding the method that should be used to screen for infection in recipients and donors^50^. Serological tests are usually very sensitive, but antibody responses usually persist for an extended period after treatment^48^ ^,^ ^51^ ^-^ ^53^; therefore, they must be carefully evaluated for use during follow-ups of transplanted patients. Nevertheless, a reduction in parasite-specific antibodies after treatment and during follow-up of transplanted patients might be indicative of parasite elimination and the absence of reinfection. Serology was only used in the series reported by Moghazy et al.^12^.

Among the dozens of diagnostic antigens that have been studied over the past four decades, one example of a detailed standardization and evaluation was reported for the microsomal antigens MAMA (*S. mansoni*) and JAMA (*S. japonicum*)^54^. These two antigens were evaluated using an in-house Falcon Assay Screening Test (FAST)-ELISA and Western Blot. Additionally, well-characterized peptides from MAMA/JAMA were investigated. Several independent research laboratories have developed and tested different recombinant peptides, which may represent an effective approach for generating new, well-standardized, and specific antibody detection systems^55^ ^-^ ^58^. Such systems avoid the need for complex antigen preparations and the inherent source of variable reactivity that complex antigen preparations can present when incubated with antibodies. However, it has also been shown that the use of assays with specific recombinant antigens or even peptides results in the loss of sensitivity of the diagnostic test^55^ ^,^ ^58^. One strategy to avoid these limitations is to use direct antigen detection systems instead of host immunoglobulins. In schistosomes, parasite-specific circulating cathodic antigens (CCA) and circulating anodic antigens (CAA), can be detected in biological fluids during late-patency, pre-patency, and early-patency^59^ ^-^ ^61^. For CCA, a point-of-care commercial test (POC-CCA) was developed and is now available in many endemic countries^62^ ^,^ ^63^. A positive result has been reported for the detection of a CCA in the urine of a patient^38^. However, this rapid diagnostic test requires improved quality assurance from manufacturers and extensive specificity evaluations before wider use is recommended, particularly when applied to examinations of individual patients and in low- and non-endemic settings^46^ ^,^ ^64^ ^-^ ^67^. Finally, there are promising results that indicate that a serum CAA detection method may provide an accurate diagnosis of active schistosomiasis infections; however, this method is not yet ready for large-scale application^68^ ^,^ ^69^.

### ● Possible exacerbation of disease severity

After demonstrating the presence of an active infection, the potential for increased morbidity owing to long-term immunosuppressive therapy must be considered. In contrast to other infectious agents (e.g., protozoan parasites and bacteria), *Schistosoma* larvae and worms do not replicate or remain in the dormant stage in vertebrate hosts, and eggs containing miracidia usually die within 1-2 weeks after deposition in host tissues. Consequently, “reactivation” and “hyperinfection” (Table 2) do not occur in schistosomiasis. However, many reports and reviews have incorrectly identified the latter as a main concern for patients with transplants. Other terms have also been used to indicate the risk of increased morbidity, but sometimes without the clear distinction, as shown in Table 2. Briefly, “reactivation” can be defined as upregulated metabolism and activity of infectious agents, particularly after a period of latency or low metabolism and reproduction. Meanwhile, “recrudescence” or “relapse” indicates the return of disease manifestations, while “recurrence” refers to a more general concept indicating a return or “coming back” of disease (Table 2). Recrudescence of disease, not necessarily reactivation of infection, is the main concern when considering the risk of liver graft loss associated with schistosomiasis.

**TABLE 2:** Glossary.

**Recurrence**	A more general concept of “coming back”, returning
**Recrudescence (Relapse)**	A more specific concept of a recurrent deleterious process, disease; exacerbation of lesions or symptoms; **example**: returning clinical manifestations of malaria from “reactivation” of liver hypnozoites.
**Reactivation**	Infectious agents that increase metabolism and reproduction activities, after being in a latent or dormant stage; **examples**: *Toxoplasma gondii*, *Trypanosoma cruzi*.
**Hyperinfection**	*De novo* infection superimposed on an existing infection; **example**: *Strongyloides stercoralis* and the acceleration of infective L3 maturation while still in transit in intestinal lumen leading to successive “waves” of larvae migrating through tissues after penetration in the intestinal mucosa.

### ● Schistosomiasis and immune response to infection

Experimental infections and human studies have demonstrated that parasite migration and maturation induce a predominant type-1 early immune response associated with schistosomula control and some schistosomiasis-associated symptoms^70^ ^-^ ^72^. Adult worms successfully release their eggs into feces or urine (*S. haematobium*). However, large numbers of eggs travel through the venous circulation and are trapped in other and more remote host tissues. This can lead to progressive organ damage. Antigens released by tissue-trapped schistosome eggs can also stimulate a shift toward a type 2-biased immune response, which subsequently modulates the initial pro-inflammatory response and orchestrates granuloma formation around parasite eggs^73^ ^-^ ^75^. The granuloma reaction protects the host by limiting the cytotoxic effect(s) mediated by secreted antigens and reduces tissue damage. However, the induction of an egg antigen-centered type-2 immune response also stimulates tissue fibrosis, which may lead to portal hypertension, both of which are hallmarks of severe chronic schistosomiasis, usually developing in a small proportion of patients^76^. A chronic and progressive increase in the modulatory immune response of a host favors parasite survival and minimizes morbidity via immune-mediated inflammation^77^ ^-^ ^80^.

The balance of the immune response induced by *Schistosoma* is a determining factor for morbidity. Long-term immunosuppressive therapy can modify the natural course of the disease. However, *Schistosoma*-infected mice treated with steroids did not show significantly altered parasite loads; however, egg retention in the intestinal wall increased^81^ ^-^ ^84^. Pyrro et al.^84^ also observed reduced liver granulomatous inflammation and reduced collagen deposition in mice infected with *Schistosoma* and treated with dexamethasone. A significant reduction in gamma interferon, interleukin (IL)-12, and IL-4 and an increase in IL-10 were detected in the serum. In another study, increased numbers of eggs in the host tissue and a reduction in granuloma size around the eggs were reported in rabbits infected with *S. japonicum* and treated with cortisone^85^. However, when mice were infected with *S. mansoni* and treated with immunosuppressive therapy that combined cyclosporine A and hydrocortisone, liver damage was exacerbated^86^. In contrast, a systematic review of studies examining African individuals co-infected with the human immunodeficiency virus and *Schistosoma* spp. found no evidence of increased severity of helminthic infections. The latter study also confirmed data from experimental infections that indicated a reduction in egg numbers eliminated in feces^87^. Therefore, it is hypothesized that immunosuppressive therapy, including cyclosporine, may mediate an anti-helminthic effect without a deleterious effect on granuloma formation in experimental infections^88^ ^,^ ^89^.

The immune response of a host is a key determining factor in the process of worm maturation^90^ ^-^ ^95^ and elimination of eggs into feces^96^ ^-^ ^98^. However, there is no evidence that immune-mediated mechanisms favor worm fecundity or oviposition^85^. Accumulating evidence has demonstrated that schistosomiasis is characterized by a complex inflammatory response involving a diverse set of immune components. Downregulation of certain immune mechanisms has also been demonstrated. By studying the immune modulation exerted by helminthic parasites, valuable insights into novel mechanisms of immune tolerance induction in patients with transplants have been obtained^99^ ^,^ ^100^. It should also be noted that the liver is proposed to be a naturally immunotolerant tissue^101^ ^,^ ^102^. It is not expected that immunosuppressive therapies will lead to significant changes in the pathogenesis attributable to migrating larvae and worms, and hence to increased morbidity and its manifestations. However, it is possible that immunosuppressive regimens can be tailored for LT, thereby further reducing the risk of an unbalanced immune response^103^.

Most of the pathogenesis of schistosomiasis is derived from the trapping of eggs in tissues, particularly in the intestinal/bladder wall or liver and bladder-ureter tissues. The former represents a “way out”, while the latter is a “dead end”. If treatment with immunosuppressive drugs results in increased egg production, this would lead to increased severity of schistosomiasis in patients with transplants. However, to date, there is no evidence from experimental models that supports such a mechanism^99^ ^,^ ^104^. In contrast, reductions in fecal egg elimination have been documented, which was largely attributed to the failure of the host immune response, whereby eggs were redistributed, and a greater number of eggs were trapped in tissues^83^ ^,^ ^85^ ^,^ ^86^ ^,^ ^105^ ^,^ ^106^. It is also possible that immunosuppression may negatively affect worm fecundity^91^.

Only 0.4% to 1.8% of a large series of patients with transplants had an association with schistosomiasis^8^ ^,^ ^92^ ^,^ ^93^. This rare association explains the small number of patients reported in the literature and included in this review. This limitation highlights a requirement for prospective multicenter studies with extended follow-up^4^ ^,^ ^33^. Among the 43 reported cases examined in the present study (Table 1), evidence for a role of schistosomiasis in increased morbidity and graft loss was weak. For example, the possibility was only considered for six of the patients, but without confirmation^31^ ^,^ ^33^ ^,^ ^38^ or with complete recovery from liver enzyme abnormalities^25^ ^,^ ^26^. Contrary to early stage granuloma and non-degenerated eggs^25^ ^,^ ^26^, epithelioid or calcified granulomas do not represent evidence of a significant contribution to failure of transplantation^31^ ^,^ ^38^. 

### ● Treatment

Treatment of schistosomiasis with praziquantel (PZQ) has been well established for decades (40-60 mg/kg, single dose or divided into two doses)^72^ ^,^ ^107^ ^-^ ^109^. Children benefit from a higher dose (60 mg/kg) and from a new pediatric formulation that is in the final stages of development and large-scale production^110^. Cure control is recommended 90 days after treatment in coordination with an appropriate combination of diagnostic tests. The latter issue remains to be addressed in future studies, particularly for recipients of transplants. Repeated doses of PZQ are guided by adequate diagnosis of infection activity (e.g., activity-specific serology, egg detection, polymerase chain reaction, or antigen detection). In two instances (Table 1), very early (15 days) and excessive repetitions of PZQ treatment were conducted on days 2, 3, 18, and 42 post-transplantation. One rationale for repeating PZQ is that the drug does not affect larvae or young worms that can eventually escape the first round of treatment. However, this does not modify the usual protocol for cure control after 90 days, as there is no requirement for urgent intervention. Furthermore, the inability to demonstrate increased morbidity or graft loss weakens the consideration of any “blind” or “preventive” treatment.

### ● Recommendations for screening and management

**FIGURE 1:**
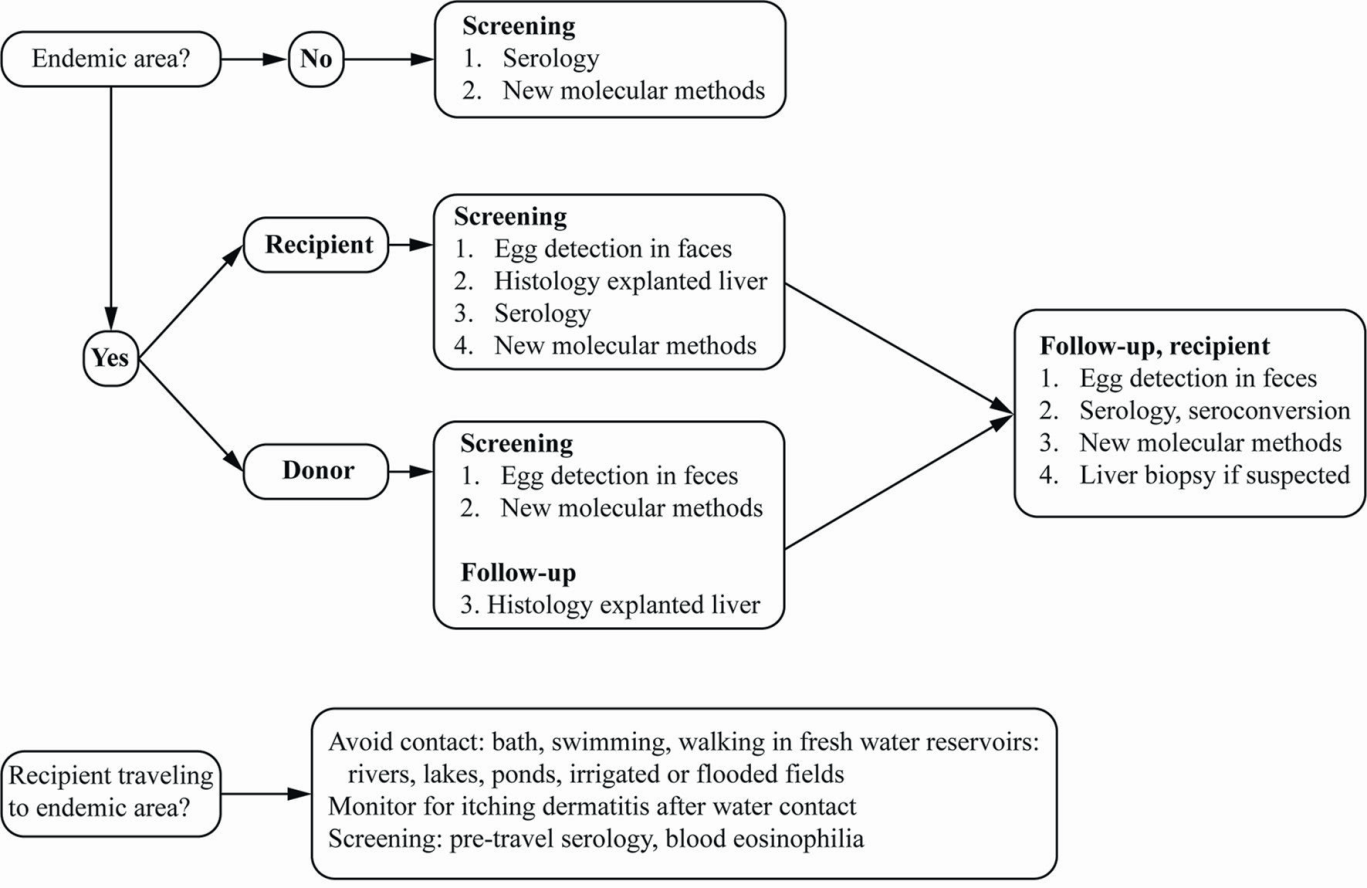
Recommendations for management of donors and recipients of liver transplants associated with *Schistosoma* species affecting the liver. Modified from Clemente et al.^111^, La Hoz and Morris^112^. See updated mappings of endemic areas^23^ ^,^ ^113^. Recipients and donors from non-endemic areas may be screened with serology or any validated DNA or antigen detection tests under development and performance evaluations. Please see updated list of available tests at Global Schistosomiasis Alliance website^116^. Egg detection should be performed using methods that are more sensitive than the Kato-Katz method. These include: the Lutz method (Hoffman, Pons & Janer - HPJ, spontaneous sedimentation of 1 g feces), the Ritchie method (centrifugation after ethyl-acetate treatment of 1 g feces), and magnetic isolation (Helmintex), the most sensitive egg-detection method available^117^. Rectal biopsy is recommended when parasitological, immunological, and molecular methods are negative in a suspected schistosomiasis. Serology is not confirmatory, but seroconversion provides a higher degree of suspicion. Whenever eggs are detected in stools or at histology, treatment with a single 40-60 mg/kg dose of praziquantel (PZQ) is recommended. A PZQ prescription without confirmatory egg detection (e.g., positive serology) remains an ongoing and controversial topic for discussion.

Figure 1 summarizes the provisional recommendations for management of donors and recipients from endemic and non-endemic areas for screening and follow-up (adapted from Clemente et al.^111^; La Hoz and Morris^112^). Blood eosinophilia is not a reliable marker for chronic schistosomiasis but it is for acute schistosomiasis, particularly in endemic areas^71^. However, blood eosinophilia may assist in screening patients from non-endemic areas^113^ ^,^ ^114^. Rectal biopsy may also be an option for egg detection, but only after repeated examinations with parasitological or serological methods more sensitive than the Kato-Katz method. Figure 1 also presents the recommended preventive actions for patients with LT travelling to endemic areas. (For an updated list of countries endemic for schistosomiasis, see WHO 2022)^115^.

## CONCLUDING REMARKS

Isolated case reports and small patient series are not ideal conditions for collecting strong evidence and reaching definitive conclusions, particularly due to the lack of a systematic description of all relevant information. However, the cohort of 43 reviewed patients represents the total number of published cases from the four most comprehensive databases and is the best available source for an exploratory study of a rare association.

Available data indicate that schistosomiasis is not an impeditive condition for LT; however, some controversial issues remain unresolved. Infected individuals are generally treated with PZQ, although recipients who have not received treatment have exhibited good prognosis^33^. Treatment with PZQ should be performed only if viable eggs are detected. The immunosuppressive effects that increase the morbidity of schistosomiasis in patients with an LT have not been well characterized. Therefore, careful follow-up of schistosomiasis-infected grafts and recipients in accordance with well-designed research protocols and adequate diagnostic tools is urgently required. This review provides a valuable starting point for collaborative efforts to establish improved protocols for the investigation and follow-up of patients with transplants. According to this review, the use of liver grafts with schistosomiasis appears to be safe, and infections detected in donors or recipients should not prevent transplantation.
